# Volumetric quantification identifies some left atrial dilations undetected by left atrium:aorta ratio measurements: A prospective echocardiographic study in 155 Cavalier King Charles Spaniels with and without degenerative mitral valve disease

**DOI:** 10.1371/journal.pone.0300827

**Published:** 2024-03-28

**Authors:** Valérie Chetboul, Camille Poissonnier, Pierre Foulex, Maria Paz Alvarado, Émilie Trehiou-Sechi, Vittorio Saponaro, Peggy Passavin, Loïc Desquilbet

**Affiliations:** 1 École Nationale Vétérinaire d’Alfort, CHUVA, Unité de Cardiologie d’Alfort (UCA), Maisons-Alfort, France; 2 INSERM, IMRB, Univ Paris Est Créteil, Créteil, France; 3 Department of Biostatistics and Clinical Epidemiology, École Nationale Vétérinaire d’Alfort, Maisons-Alfort, France; University of Alberta, CANADA

## Abstract

**Introduction:**

Degenerative mitral valve disease (DMVD) is the most common canine heart disease with a high predisposition in Cavalier King Charles Spaniels (CKCSs). Mitral regurgitation related to DMVD can lead to left atrial (LA) dilation, which is associated with survival time. Left-atrial-to-aortic (LA:Ao) ratio assessed by two-dimensional echocardiography is commonly used to evaluate LA size. The objectives of this prospective observational study were therefore 1) to compare different echocardiographic methods (i.e., monoplane and biplane Simpson’s methods of discs (SMOD) and area-length methods (ALM)) in evaluating LA volume (LAvol) in CKCSs, 2) to assess LA volumes according to DMVD severity and, 3) compare the ability of LAvol and LA:Ao ratio to identify LA enlargement in CKCSs with subclinical DMVD (i.e., American College of Veterinary Internal Medicine (ACVIM) stage B).

**Materials and methods:**

155 CKCSs, either healthy or affected by DMVD, were recruited. Variability and concordance between volumetric methods were evaluated. Values were analyzed according to 2019 ACVIM stages.

**Results:**

All Lin’s concordance correlation coefficients regarding intra- and inter-observer variability were considered as very good to excellent. Monoplane methods and ALM produced higher values of LAvol than biplane methods and SMOD, respectively. The upper limit of normal end-systolic LAvol/body weight (LASvol/BW) was defined as 0.90 mL/kg. Left atrial volumes significantly increased with ACVIM stages. Additionally, 37% of stage B1 CKCSs demonstrated LA enlargement using LASvol/BW assessment, with significantly lower LASvol/BW values in dogs with regurgitation fraction ≤30% than in others (*p*<0.01).

**Conclusion:**

In CKCSs, LAvol methods are not interchangeable. In ACVIM stage B CKCSs, LAvol quantification is more effective to detect LA enlargement than LA linear measurements.

## Introduction

Degenerative mitral valve disease (DMVD) is the most common acquired canine heart disease with a high predisposition in small to medium sized dog breeds, and specifically the Cavalier King Charles Spaniel (CKCS) breed [1–5]. Mitral valve (MV) lesions associated with DMVD are characterized by progressive myxomatous degeneration of the leaflets with thickened and elongated *chordae tendineae*, resulting in incomplete leaflet apposition during systole, and secondary mitral regurgitation (MR) [6]. Progression of the disease is associated with MR worsening, potentially leading to an overload of the left heart volume (left atrial (LA) dilation, followed by left ventricular (LV) enlargement), with left-sided congestive heart failure (CHF), which occurs in advanced DMVD stages [7, 8]. Echocardiographic assessment of the disease is based on the evaluation of LA and LV dimensions [9, 10], and the LA diameter is one of the strongest predictors of clinical outcome [11, 12]. The LA diameter is usually measured on the right parasternal transaortic short-axis view and compared to the aortic (Ao) diameter (LA:Ao ratio) [7, 8, 13, 14]. However, LA dilation can develop in medio-lateral, cranio-caudal, or ventro-dorsal directions. Therefore, the LA:Ao ratio may not be reliable for the early detection of LA dilation. In addition, LA volume measurements recently have been reported in the dog using monoplane and biplane Simpson’s methods of discs (SMOD) and area-length methods (ALM) [15–21], and a certain degree of discrepancy has been observed between these volumetric methods and LA:Ao ratio values in identification of LA enlargement [16]. To the best of the authors’ knowledge, these echocardiographic methods of LA size assessment have not been specifically evaluated in a large population of CKCSs both healthy and affected by DMVD and their ability to detect early LA enlargement in this specific breed remains unknown.

The objectives of this prospective observational study were therefore 1) to compare different echocardiographic methods in evaluating LA volumes, i.e., monoplane and biplane SMOD and ALM, in a large population of CKCSs, 2) to assess LA volumes according to DMVD severity and, 3) to compare the ability of LA volumes and LA:Ao ratio in identifying LA enlargement in CKCSs with subclinical DMVD (i.e., stage B of the American College of Veterinary Internal Medicine (ACVIM) classification).

## Materials and methods

### Study population

The study population consisted of adult (age > 12 months) client-owned CKCSs, either healthy (ACVIM stage A [8]) or affected by DMVD (ACVIM stages B to D [8]), as determined by clinical, radiographic and echocardiographic examinations performed by four trained observers with at least 7 years of experience from the Alfort Cardiology Unit (UCA) at the National Veterinary School of Alfort (France), that were prospectively recruited between 2017 and 2019. All equivocal data or images (i.e., dogs with normal LA:Ao ratios but increased LA volumes) were reviewed by a board-certified cardiologist (VC).

Dogs were not included if they were suffering from any other hemodynamically significant heart diseases, or any systemic diseases, or if they were dehydrated. Nor were included dogs with overt respiratory clinical signs at time of presentation and during physical examination. The presence of arrhythmias was not an exclusion criterion.

### Epidemiological and clinical features

Data regarding age, sex, and body weight (BW) were recorded. In all dogs with DMVD, the left apical systolic heart murmur grade was also recorded by using the modified Levine 6-level classification scheme. Other clinical signs were noted if present, e.g., exercise intolerance, cough, syncope, dyspnea, and ascites.

### Echocardiography and Doppler examination

Standard two-dimensional (2D), M-mode, and Doppler examinations were performed in awake standing dogs with continuous ECG monitoring by trained observers using ultrasonographic units (Vivid 7, and Vivid E9, General Electric Medical System, Waukesha, Wisc, USA) as previously described [22]. Measurements were performed at the time of images acquisition.

#### Left atrial and ventricular diameters

Aortic and LA diameters were measured at both end-diastole [23] and end-systole [14] by 2D method using the right parasternal transaortic short axis view, and corresponding LA:Ao ratios were calculated. As CKCSs seem to have rather small end-diastolic and end-systolic LA dimensions than most other canine breeds [14, 17, 23], LA:Ao cut-off values used for defining LA enlargement were based on studies solely dedicated to CKCSs [14, 23], instead of the commonly used end-systolic cut-off value of 1.6 [8, 24]. The LA:Ao ratio was therefore considered as increased for values >1.0 at end-diastole [23] and/or ≥1.4 at end-systole [14]. Left ventricular measurements (LV end-diastolic and end-systolic internal diameters) were obtained using the 2D-guided M-mode as recommended [25], and the LV fractional shortening was calculated accordingly. For each M-mode echocardiographic variable, a mean of 3 measurements was determined from 3 consecutive cardiac cycles in the same frame and were compared with published reference ranges according to BW in the CKCS breed [23].

#### Left atrial volume evaluation

**Fig 1:**
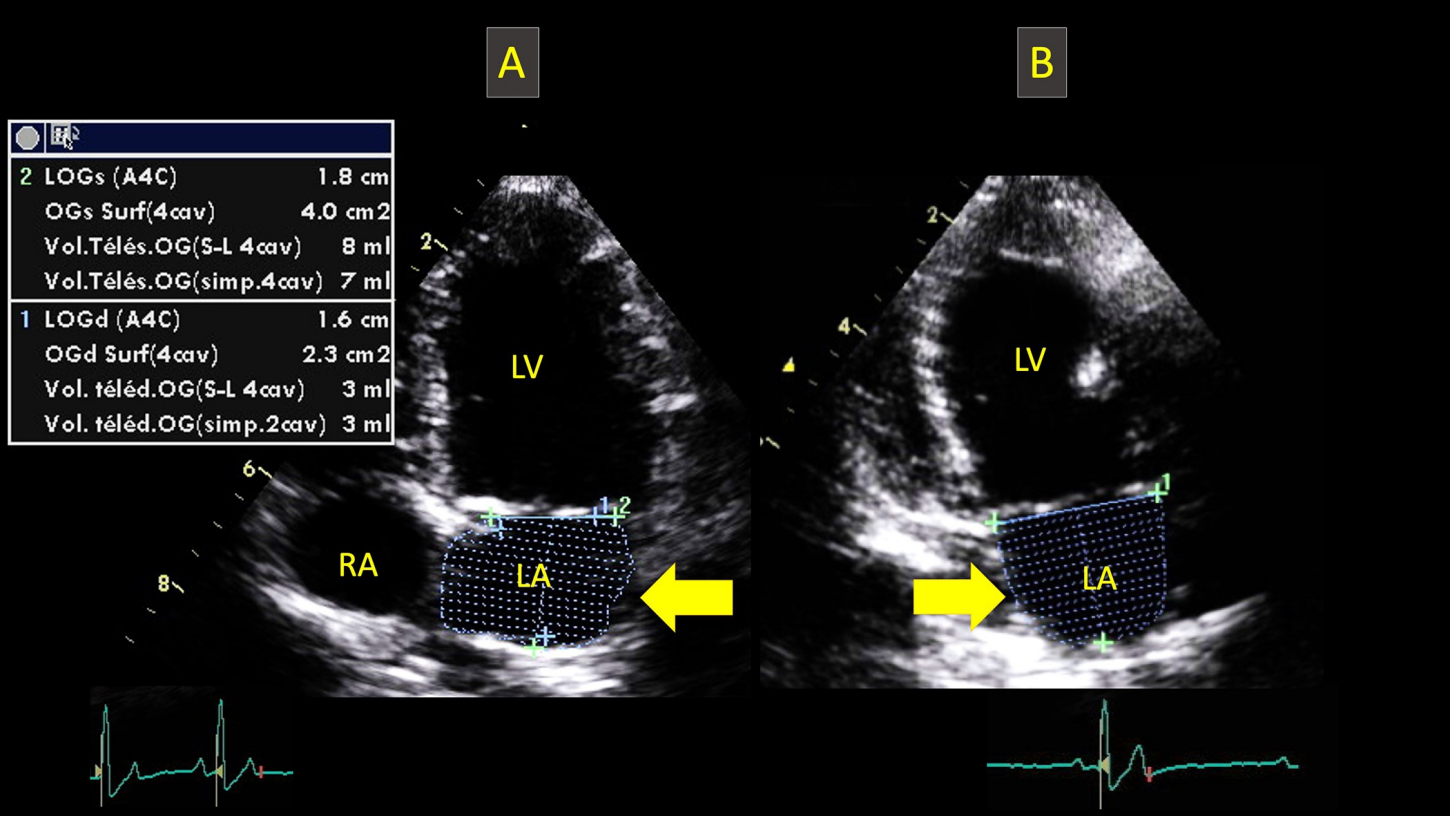
Representative measurements of the end-systolic left atrial volume (arrows) in one Cavalier King Charles Spaniel from the study, using the biplane Simpson’s method of discs and area length method from the left apical 4-chamber view (A) and 2-chamber view (B). LA: left atrium; LV: left ventricle. RA: right atrium.

Left atrial volumes were calculated at end-systole (LASvol) and end-diastole (LADvol) using the left apical 4- and 2-chamber views, whose quality had to be good in order to provide a clear delimitation of the LA endocardial border. End-systole was defined as the frame immediately before MV opening and end-diastole as the frame obtained at the time of MV closure. The endocardial border of the LA was traced at end-systole and end-diastole to obtain LA areas from both phases, with left auricle and pulmonary veins excluded from the measurements, as previously described [15–18]. The delineation between LA and LV was a straight line drawn from each hinge point across the MV annulus. Left atrial volumes were automatically calculated using SMOD and ALM with a specific software (Echopac, GE Medical Systems, Waukesha, WI, USA), as previously described [15–18] (**Fig 1**). For monoplane SMOD and ALM, LA volumes were evaluated using the left apical 4-chamber view. Regarding ALM, LA volumes were calculated according to the following formula: [0.85 x LA area ^2^]/LA length [17]. For biplane SMOD and ALM, LA volumes were calculated by averaging the LA volumes assessed from the left apical 4- and 2-chamber views, by using the corresponding methods. All LA volumes were then indexed to BW. Therefore, four echocardiographic methods were used to assess LASvol and LADvol, i.e., biplane SMOD, biplane ALM, monoplane SMOD, and monoplane ALM, thus providing 8 values for LA volumes (4 at end-systole and 4 at end-diastole). Lastly, the LA ejection fraction (EF_LA_) was calculated using the formula: 100*(LASvol–LADvol)/ LASvol for both biplane ALM and SMOD.

#### Doppler examination

In DMVD dogs with MR adequate for quantification, MR severity was evaluated using the proximal isovelocity surface area method (PISA), as previously described and validated [26]. The studied PISA variable was the regurgitation fraction (RF), defined as the percentage of stroke volume ejected into the LA during systole, and MR was considered as mild for RF ≤30%, moderate for RF >30% but ≤50%, and severe for RF >50% [27].

Peak early (E) and late (A) diastolic mitral flow velocities were determined using pulsed-wave Doppler mode from the left apical 4-chamber view. The left apical 4-chamber view optimized for the right cardiac chambers was used for color-flow Doppler examination of the tricuspid flow. When tricuspid regurgitation was identified, the peak systolic tricuspid regurgitation velocity was measured using continuous-wave Doppler mode, and the systolic right ventricle-to-right atrium pressure gradient, i.e., tricuspid regurgitation pressure gradient (TRPG) was calculated using the modified Bernoulli equation.

### Classification

Dogs were classified according to the 2019 ACVIM consensus statement on diagnosis and treatment of DMVD [8]. Stage A included healthy CKCSs without any identifiable structural heart disease and any heart murmur. Stage B (or DMVD compensated group) included CKCS dogs with DMVD, but without any past or current CHF signs. Stage B was then sub-divided into stages B1 and B2, with some CKCS breed-specific adjustments for stage B2. According to the ACVIM guidelines, stage B2 should include asymptomatic dogs with DMVD causing MR severe enough to result in cardiac remodeling (LA and LV enlargement) sufficient to recommend treatment (pimobendane) before the onset of clinical signs based on the results of a prospective clinical trial, i.e., the EPIC study [24]. This is why, in these guidelines, the same multi-breed cut-offs as those used in the EPIC study are recommended, i.e., end-systolic LA:Ao ≥1.6 and LV end-diastolic internal diameter normalized to bodyweight ≥1.7 [8]. However, these multibreed echocardiographic ACVIM cut-offs do not correctly fit with the CKCS breed regarding both LA and LV sizes [14, 23]. Therefore, in the present study, CKCS breed-specific reference intervals (instead of the ACVIM multi-breed cut-offs) were used to define LA and LV enlargement, as explained above. Stage B1 included asymptomatic dogs with mitral regurgitation that was not severe enough to meet stage B2 criteria. Stage C included dogs with past or current CHF as defined above, and stage D referred to dogs with end-stage DMVD refractory to standard CHF therapy (dogs from ACVIM stages C and D representing the decompensated DMVD group). Left-sided CHF was defined as the presence of pulmonary edema (assessed by clinical signs (e.g., cough, dyspnea) and thoracic radiographs) due to severe DMVD as confirmed by echocardiographic evidence of moderate to severe LA enlargement and MR calculated by the PISA method, as described above.

### Repeatability and inter-observer reproducibility of LA volumes and LA:Ao ratios

Repeatability (i.e., intra-observer variability) and inter-observer reproducibility were determined for LASvol/BW and LADvol/BW assessed by each method described above (i.e., monoplane and biplane ALM and SMOD, n = 8 methods), as well as for LA:Ao ratio at end-diastole and end-systole, using Lin’s concordance correlation coefficient (CCC) as well as Bland and Altman analyses, including bias and 95% limits of agreement [28–32].

The cine-loop recordings of 25 CKCSs (ACVIM stages A and B) were selected to be subjected to two analyses in a random order on two non-consecutive days over a one-week period by a single trained observer (Observer 1, VC, Dipl. ECVIM-CA Cardiology), to assess repeatability of all parameters [30]. These recordings of at least 3 consecutive cardiac cycles were also analyzed by a second trained observer (Observer 2, CP), with a 7-year experience of echocardiography and trained by Observer 1, to assess inter-observer reproducibility. Thus, a total of 750 cine-loop examinations (50 for Observer 1 and 25 for Observer 2), corresponding to 750 measurements, were performed.

Lin’s CCC was considered as unacceptable, poor, mediocre, satisfactory, fairly good, very good, and excellent when respectively <0.50, comprised between 0.51–0.60, 0.61–0.70, 0.71–0.80, 0.81–0.90, and 0.91–0.95, and >0.95 [33]. Regarding Bland and Altman analyses, repeatability and inter-observer reproducibility were considered by the clinicians as good if the 95% limits of agreement were within a +/- 0.30 mL/kg interval for LA volumes and +/- 0.2 interval for LA:Ao ratios, and if the bias was <0.25 mL/kg for LA volumes and <0.15 for LA:Ao ratios. These criteria to define a good repeatability and inter-observer reproducibility were chosen prior to the echocardiographic measurements by Observer 1 (Dipl. ECVIM-CA Cardiology) based both on her echocardiographic experience and on previously published maximal between-day standard deviations for LA/Ao ratio [22].

### Statistical analyses

Statistical analyses were performed by a computer software (Xlstat-Biomed, Version 2016, Addinsoft, Data Analysis and Statistical Solution for Microsoft Excel, Paris, France). Categorical data are reported as proportions or percentages, and continuous data as medians (interquartile ranges). Associations between ACVIM stage and epidemiological, clinical, and echocardiographic data were done using either Chi-square or Fischer’s exact tests for categorical data (sex, absence/presence of clinical signs such as cough, exercise intolerance, dyspnea, syncope, ascites) and using Kruskal-Wallis test for continuous data (age, weight, echocardiographic variables). There was no two pairwise comparison between two ACVIM stages. Correlations between LA volumes/BW and other echocardiographic variables (LA:Ao ratios, fractional shortening, RF, mitral E/A ratio, EF_LA_, and TRPG) were examined by Pearson correlation analysis. A Bland-Altman analysis was applied to investigate the concordance between the four echocardiographic methods used to assess LASvol/BW, LADvol/BW and EF_LA_ as the following: 1) concordance between biplane SMOD and biplane ALM for the assessment of LASvol/BW, LADvol/BW, and EF_LA_ (3 analyses); 2) concordance between the two monoplane methods (ALM and SMOD) for the assessment of LASvol/BW and LADvol/BW (2 analyses), and lastly, 3) concordance between monoplane and biplane ALM as well as between monoplane and biplane SMOD for the assessment of both LASvol/BW and LADvol/BW (4 analyses). In total, 9 Bland-Altman analyses were thus performed. The level of significance was set at *p* <0.05.

## Results

### General characteristics of the study population (n = 155; Table 1)

**Table 1:** Epidemiological and clinical features as well as main standard echocardiographic and Doppler variables (median (IQR), [range] and percentages) assessed in 155 Cavalier King Charles Spaniels, divided into ACVIM stage A (n = 27), stage B1 (n = 94), stage B2 (n = 15), and stages C and D (n = 19) [8].

			Whole population (n = 155)	ACVIM Stage A (n = 27)	ACVIM Stage B1 (n = 94)	ACVIM Stage B2 (n = 15)	ACVIM Stage C and D (n = 19)		*P*-value
**Sex**		*Female*	52% (81/155)	52% (14/27)	56% (53/94)	47% (7/15)		37% (7/19)	0.45
**Age (years)**			7.8 (5.7–9.2) [1.2–14.5]	4.7 (3.0–6.0) [1.2–9.1]	7.9 (6.1–9.1) [2.2–14.4]	9.1 (8.4–11.0) [6.2–14.5]		9.6 (8.5–11.3) [4.3–12.7]	<0.01
**Body weight (kg)**			9.1 (7.8–10.3) [5.0–16.5]	8.8 (6.8–10.1) [5.9–12.6]	9.2 (8.0–10.4) [5.0–16.5]	8.4 (7.8–9.4) [6.8–11.0]		9.2 (8.2–9.8) [6.1–12.0]	0.57
**Clinical signs**	*Heart murmur grade*				3 (2–4) [1–5]	5 (4–5) [4–5]		5 (5–5) [4–5]	<0.01
	*Cough*		23% (36/155)	0% (0/27)	19% (18/94)	27% (4/15)		74% (14/19)	<0.01
	*Exercise intolerance*		14% (22/155)	0% (0/27)	16% (15/94)	20% (3/15)		21% (4/19)	0.05
	*Dyspnea*		8% (12/155)	0% (0/27)	0% (0/94)	0% (0/15)		63% (12/19)	<0.01
	*Syncope*		1% (2/155)	0% (0/27)	1% (1/94)	0% (0/15)		5% (1/19)	0.42
	*Ascites*		1% (2/155)	0% (0/27)	0% (0/94)	0% (0/15)		11% (2/19)	<0.01
**Echocardiography**	*End-systolic left atrium*:*aorta ratio*		1.11 (0.99–1.35) [0.50–3.69]	1.02 (0.90–1.12) [0.77–1.36]	1.06 (0.97–1.20) [0.50–1.40]	1.65 (1.55–1.72) [1.43–1.98]		2.06 (1.83–2.77) [1.50–3.69]	<0.01
	*End-diastolic left atrium*:*aorta ratio*		0.80 (0.70–0.96) [0.45–2.73]	0.73 (0.67–0.79) [0.49–0.98]	0.76 (0.68–0.85) [0.45–1.00]	1.18 (0.96–1.32) [0.80–1.55]		1.51 (1.40–2.03) [1.24–2.73]	<0.01
**Doppler examination**	*Tricuspid regurgitation pressure gradient (mmHg)*		25 (16–39) [3–153] (n = 145)	15 (11–18) [3–35] (n = 25)	23 (18–35) [5–55] (n = 87)	34 (25–41) [16–83] (n = 14)		49 (48–64) [31–78] (n = 19)	<0.01
	*Mitral E velocity (m/s)*		0.90 (0.76–1.10) [0.40–2.11] (n = 151)	0.72 (0.63–0.85) [0.57–0.99] (n = 27)	0.87 (0.76–1.02) [0.40–1.50] (n = 91)	1.23 (1.13–1.48) [1.05–1.79] (n = 15)		1.78 (1.61–1.95) [1.20–2.11] (n = 18)	<0.01
	*Regurgitation fraction (%)*		36 (22–52) [2–77] (n = 100)	-	26 (16–36) [2–56] (n = 66)	53 (44–59) [33–72] (n = 15)		65 (53–70) [42–77] (n = 19)	<0.01

During the study period, a total of 155 CKCSs either healthy (27/155, 17%) or affected by DMVD (128/155, 83%) were recruited (BW = 9.1 kg [7.8–10.3]; male-to-female ratio = 0.91; age = 7.8 years [5.7–9.2]).

### ACVIM classification at the time of diagnosis for the whole study population (n = 155) and clinical features of dogs with DMVD (n = 128; Table 1)

According to the 2019 ACVIM classification [8], 27/155 dogs (17%) were considered as healthy (stage A), 94/155 (61%) and 15/155 (10%) were in stages B1 and B2, respectively, 19/155 (12%) had past or current CHF, with 18/155 in stage C and only 1 in stage D. Cardiac auscultation revealed a left apical systolic heart murmur in all dogs with DMVD, with a median grade of 3/6 (1–4). Among DMVD dogs, 71/128 (55%) received one or more treatment at admission. Treatments included furosemide (n = 17/128, dosage = 1.90 mg/kg/day [1.10–2.72]), benazepril (n = 63/128, dosage = 0.28 mg/kg/day [0.25–0.38]), pimobendan (n = 23/128, dosage = 0.34 mg/kg/day [0.26–0.47]), and spironolactone (n = 32/128, dosage = 1.75 mg/kg/day [1.00–2.50]). Out of the 93 B1 DMVD dogs, 34 (37%) were already receiving benazepril, 16 (17%) spironolactone and/or 8 (9%) pimobendan prescribed by referring practitioners.

### Echocardiographic and Doppler findings

#### General echocardiographic and Doppler features (Table 1)

In healthy CKCS dogs (ACVIM stage A), the median LA:Ao ratios were 0.73 (0.67–0.79) at end-diastole and 1.02 (0.90–1.12) at end-systole. End-systolic and end-diastolic LA:Ao ratios were different between ACVIM stages (*p* <0.01).

The PISA method could be applied to 100/128 (78%) CKCS with DMVD. The RF was higher (*p* <0.01) for dogs from the ACVIM decompensated stages than for those in stage B.

Tricuspid regurgitation adequate for TRPG assessment was identified in 145/155 (94%) CKCSs, and pulmonary arterial hypertension (defined as TRPG >46 mmHg [34]) was diagnosed in 22 DMVD dogs: 5/87 (6%) in ACVIM stage B1, 2/14 (14%) in ACVIM stage B2, 15/19 (79%) in ACVIM stages C and D. Four out of the 5 ACVIM DMVD B1 dogs with moderate PAH did not show overt respiratory clinical signs at time of DMVD diagnosis and inclusion. One had history of mild tracheal collapse on previous thoracic radiographs and for the three others, snoring during sleep at home was the sole clinical sign reported by the owners. The other B1 dog was obese with at least a 3-kg overweight. Therefore, pulmonary hypertension for these 5 ACVIM B1 dogs was consistent with at least type 3 PAH according to the ACVIM classification [34]. One ACVIM stage B1 dog (see Table 1) with a high TRPG gradient (52 mmHg) underwent a single syncope episode after an intense physical effort.

A significant correlation was found between RF and the end-diastolic (r = 0.73, *p* <0.01), and end-systolic (r = 0.66, *p* <0.01) LA/Ao ratios, between RF and TRPG (r = 0.65, *p* <0.01), and lastly between both the end-diastolic (r = 0.57, *p* <0.01) and end-systolic (r = 0.48, *p* <0.01) LA/Ao ratios and TRPG. Additionally, RF, end-diastolic and end-systolic LA:Ao ratios, TRPG, fractional shortening, and mitral E velocity were significantly different between ACVIM stages (*p* <0.01 for all parameters).

Regarding ACVIM stage B dogs, RF, end-diastolic and end-systolic LA:Ao ratios, TRPG, and mitral E velocity were significantly higher in B2 DMVD dogs than in B1 DMVD dogs (*p* <0.01). Among the B1 DMVD dogs, those with a RF >30% had a significantly higher end-systolic LA:Ao ratio, mitral E velocity, and TRPG than dogs with a RF ≤30% (*p* <0.01).

#### Left atrial volumes according to ACVIM stages—comparison with other echocardiographic variables

**Fig 2:**
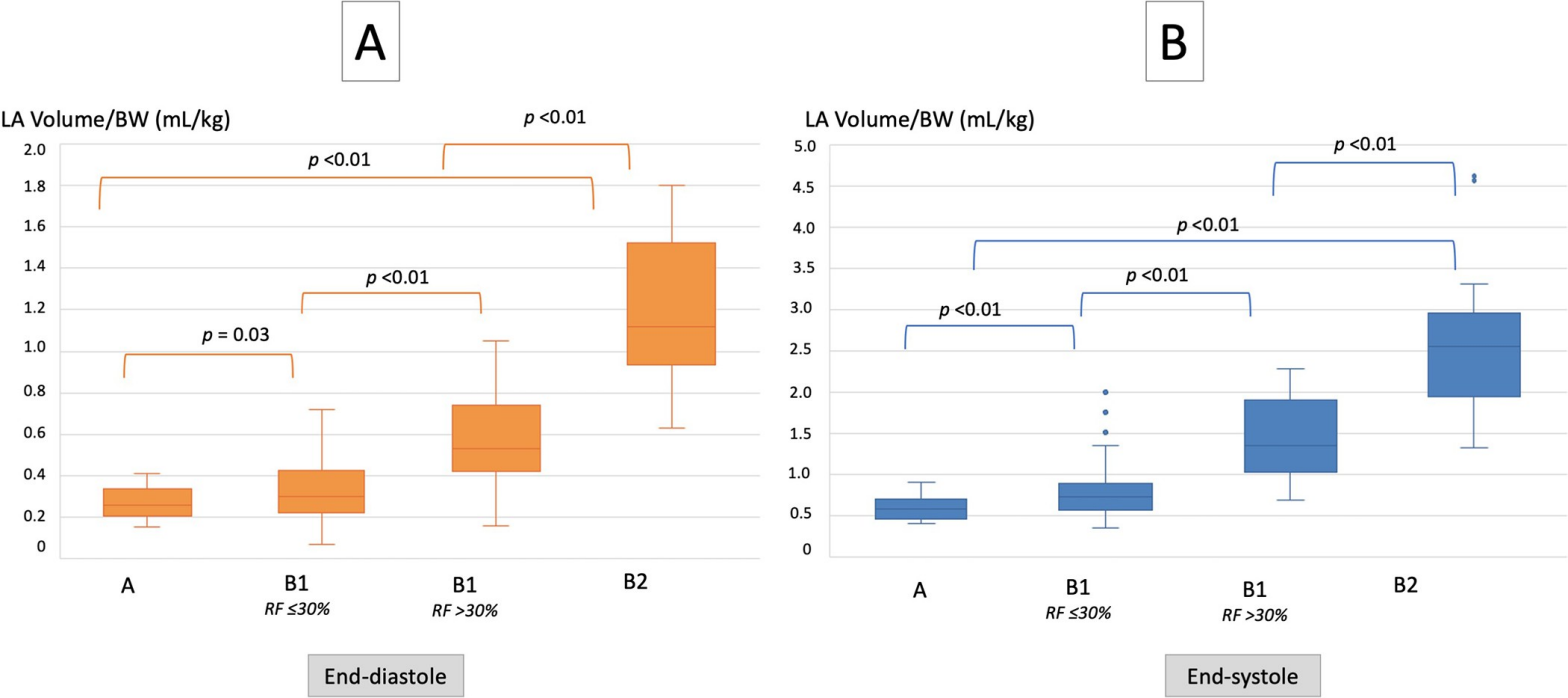
Box plots illustrating end-diastolic (A) and end-systolic (B) left atrial (LA) volumes normalized to body weight (BW) assessed by the biplane Simpson’s method of discs in the 133 Cavalier King Charles Spaniels from the study belonging to ACVIM stages A (n = 27) and B (n = 106; 92 in stage B1 and 14 in stage B2) for which LA volume/BW values were available. *RF*: *regurgitation fraction*.

Left atrial volumes indexed to BW (LASvol/BW and LADvol/BW) assessed by each echocardiographic method, are presented in **Table 2.** Values of LASvol/BW and LADvol/BW, calculated by monoplane and biplane ALM and SMOD, increased with ACVIM stages (*p* <0.01; **Fig 2**) and were also correlated with both end-systolic and end-diastolic LA:Ao ratios, respectively (*p* <0.01). Similar significant correlations were found between LASvol/BW and LADvol/BW, assessed by monoplane and biplane ALM and SMOD, and RF, TRPG, and mitral E velocity (*p* <0.01).

**Table 2:** Left atrial volumes and ejection fraction (median [interquartile range]) assessed in 155 Cavalier King Charles Spaniels using the monoplane and biplane area length methods (ALM) and the Simpson’s methods of discs (SMOD) from the left apical 4- and 2-chamber views, according to the ACVIM classification [8].

	Total	ACVIM stage A	ACVIM stage B1	ACVIM stage B2	ACVIM stages C and D	*P*-value
	(n = 155)	(n = 27)	(n = 94)	(n = 15)	(n = 19)	
**Monoplane methods (4-chamber view)**						
*LASvol/BW (mL/kg)—SMOD*	0.95 [0.66–1.79]	0.61 [0.46–0.71]	0.91 [0.69–1.39]	2.65 [2.05–3.23]	5.86 [4.02–7.43]	<0.01
	n = 150	n = 27	n = 92	n = 14	n = 17	
*LASvol/BW (mL/kg)—ALM*	0.95 [0.68–1.74]	0.62 [0.55–0.80]	0.93 [0.70–1.44]	3.18 [2.25–3.79]	5.67 [3.86–7.17]	<0.01
	n = 104	n = 19	n = 66	n = 11	n = 8	
*LADvol/BW (mL/kg)—SMOD*	0.47 [0.28–0.83]	0.26 [0.21–0.34]	0.43 [0.29–0.62]	1.53 [1.04–1.81]	3.28 [2.14–4.86]	<0.01
	n = 150	n = 27	n = 92	n = 14	n = 17	
*LADvol/BW (mL/kg)—ALM*	0.44 [0.32–0.83]	0.33 [0.23–0.39]	0.43 [0.30–0.66]	1.78 [1.17–2.08]	3.03 [2.00–4.61]	<0.01
	n = 103	n = 19	n = 65	n = 11	n = 8	
**Biplane methods (2- and 4-chamber views)**						
*LASvol/BW (mL/kg)—SMOD*	0.86 [0.62–1.75]	0.57 [0.47–0.68]	0.83 [0.64–1.19]	2.55 [2.04–2.82]	5.09 [2.90–5.91]	<0.01
	n = 150	n = 27	n = 92	n = 14	n = 17	
*LASvol/BW (mL/kg)—ALM*	0.87 [0.62–1.58]	0.62 [0.56–0.74]	0.86 [0.63–1.18]	2.87 [2.35–3.06]	4.76 [3.47–5.63]	<0.01
	n = 97	n = 19	n = 60	n = 11	n = 7	
*LADvol/BW (mL/kg)—SMOD*	0.39 [0.26–0.71]	0.26 [0.21–0.33]	0.35 [0.27–0.51]	1.12 [0.99–1.44]	2.90 [1.68–3.93]	<0.01
	n = 150	n = 27	n = 92	n = 14	n = 17	
*LADvol/BW (mL/kg)—ALM*	0.39 [0.28–0.70]	0.29 [0.24–0.37]	0.37 [0.28–0.55]	1.20 [1.10–1.68]	2.76 [1.76–3.87]	<0.01
	n = 96	n = 19	n = 59	n = 11	n = 7	
*Left atrial ejection fraction (%)—SMOD*	56% [48–63]	56% [48–61]	58% [50–66]	55% [49–58]	44% [35–52]	<0.01
	n = 150	n = 27	n = 92	n = 14	n = 17	
*Left atrial ejection fraction (%)—ALM*	53% [48–62]	57% [49–60]	53% [49–63]	54% [51–58]	47% [36–50]	0.04
	n = 96	n = 19	n = 59	n = 11	n = 7	

LASvol/BW: end-systolic left atrial volume normalized to body weight. LADvol/BW: end-diastolic left atrial volume normalized to body weight.

#### Left atrial volumes in ACVIM B1 dogs

**Fig 3:**
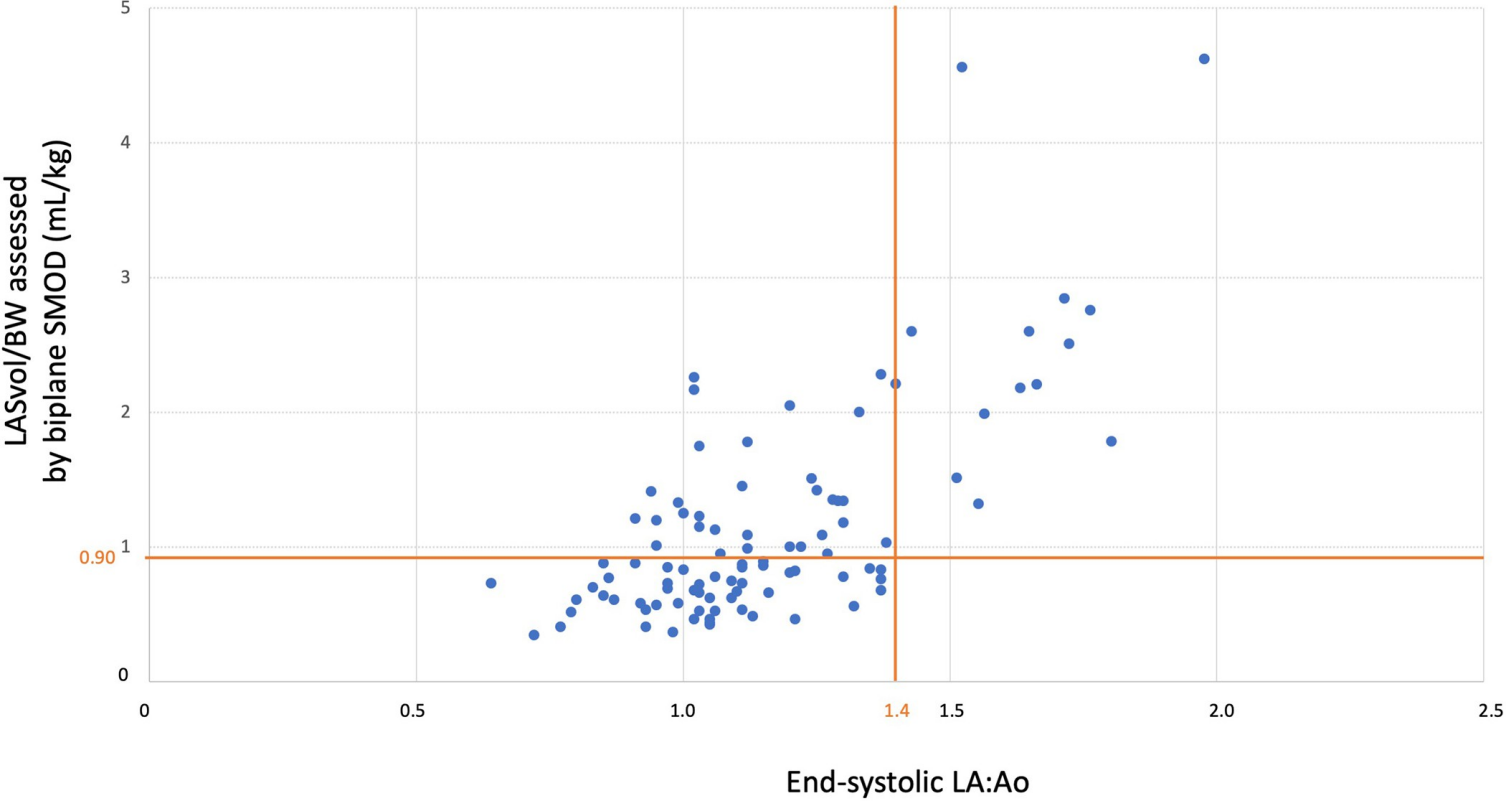
Scatterplot showing discrepancies in the diagnosis of left atrial enlargement using linear (end-systolic left atrium-to-aorta ratio (LA:Ao)) *versus* volumetric method (end-systolic left atrial volume normalized to body weight (LASvol/BW) assessed by the biplane Simpson’s method of discs (SMOD)) of left atrial size quantification in the Cavalier King Charles Spaniels from the study belonging to ACVIM stage B and for which both values were available (n = 97; 84 in stage B1 and 13 in stage B2). The threshold for identification of left atrial enlargement was 1.4 for the end-systolic LA:Ao ratio [14] and 0.9 mL/kg for LASvol/BW. Out of the 83 ACVIM B1 dogs with normal LA/Ao ratios, 31/83 (37%) showed increased LASvol/BW values despite end-systolic LA:Ao ratios within normal range (in the upper left-hand corner of the scatterplot).

In B1 DMVD dogs LASvol/BW (assessed by monoplane and biplane ALM and by monoplane and biplane SMOD; **Fig 2**) was lower (*p* <0.01) in dogs with mild MR (RF ≤30%; 0.82 mL/kg [0.64–1.05], 0.78 mL/kg [0.59–0.96], 0.79 mL/kg [0.59–1.05], and 0.73 mL/kg [0.57–0.88], respectively) than in dogs with a moderate to severe MR (RF >30%; 1.65 mL/kg [1.17–1.85], 1.31 mL/kg [1.05–1.72], 1.45 mL/kg [1.12–2.13], and 1.35 mL/kg [1.03–1.78], respectively). The 97.5^th^ percentile value of LASVol/BW in ACVIM stage A CKCSs assessed using the biplane SMOD was 0.89 mL/kg. Thus, a cut-off of 0.90 mL/kg after rounding was set as a reasonable upper normal limit for LASVol/BW assessed by using the biplane SMOD in healthy CKCSs. Out of the 83 ACVIM B1 dogs with normal LA/Ao ratios and for which both end-systolic LA:Ao ratio and LASvol/BW values were available, 31/83 (37%) showed increased LASvol/BW values (despite end-systolic LA:Ao ratios within normal range; **Fig 3**) and 19/31 (61%) also showed end-diastolic LV dilation. For these two sub-groups of ACVIM B1 CKCSs, RF values were 34% [27–42] and 38% [30–44], respectively. Similarly, out of the 91 ACVIM B1 dogs with normal LA/Ao ratios and for which end-diastolic LA:Ao ratio and LASvol/BW values were both obtained, 36/91 (40%) showed increased LASvol/BW values (despite end-diastolic LA:Ao ratios within normal range). For this sub-group of B1 dogs, RF value was 35% [27–41].

#### LA ejection fraction (Table 2)

A significant difference in EF_LA_ was observed between ACVIM stages (*p* <0.01 and 0.04 for SMOD and ALM, respectively). Finally, a negative correlation was identified between RF and EF_LA_ assessed by the biplane SMOD (r = -0.24, *p* = 0.02), but not using the biplane ALM (r = -0.13, *p* = 0.31).

#### Comparison of methods for assessing LA volumes (Figs 4 and 5)

Results of Bland and Altman analyses used to compare the different methods of LA volumes assessment are presented in **Table 3**. Biplane ALM produced higher values of LASvol/BW and LADvol/BW in comparison to biplane SMOD. Monoplane ALM also led to higher values of LASvol/BW and LADvol/BW in comparison to biplane ALM and also monoplane SMOD. Lastly, monoplane SMOD produced higher values of both LA volumes in comparison to biplane SMOD.

**Fig 4:**
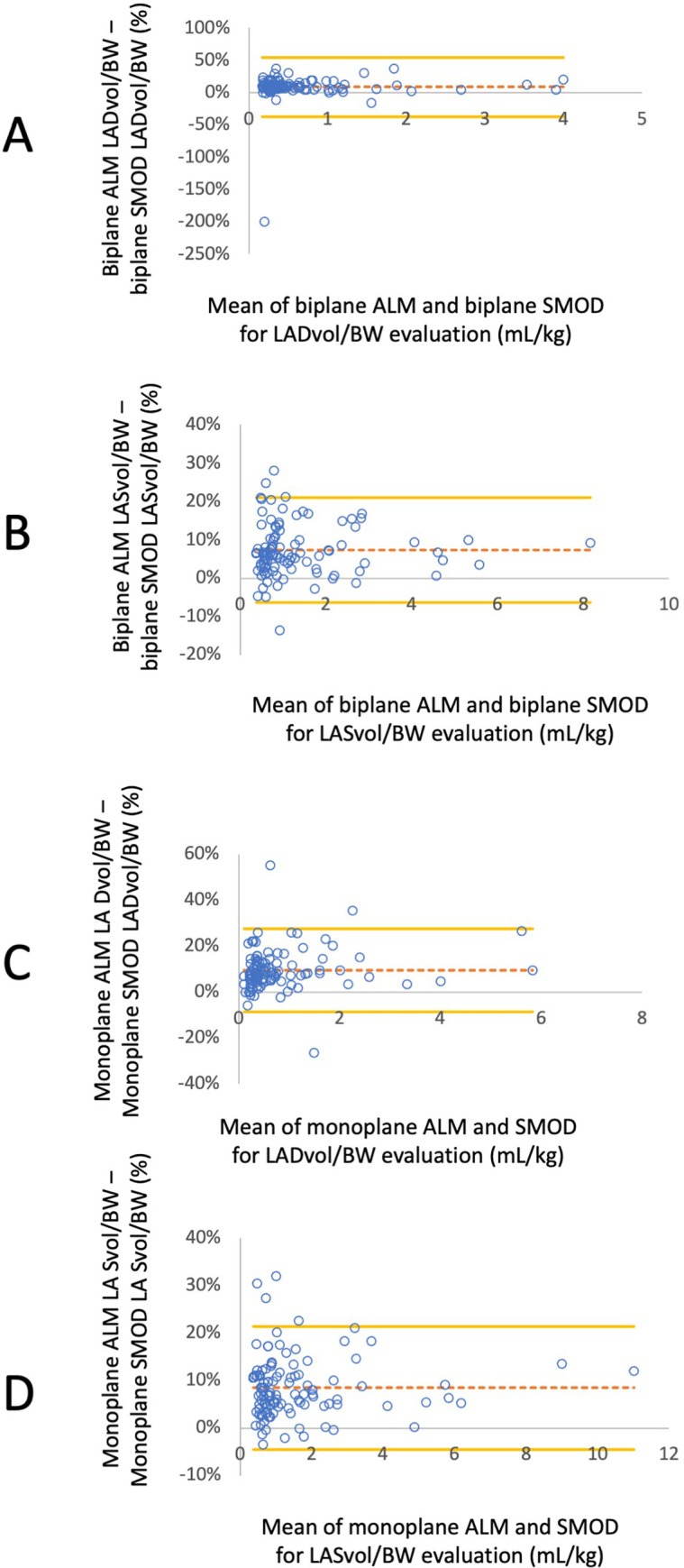
Bland-Altman analysis illustrating differences between end-diastolic and end-systolic left atrial volumes normalized to body weight (LADvol/BW and LASvol/BW, respectively) assessed by different echocardiographic methods (i.e., monoplane and biplane area length methods (ALM) and Simpson’s methods of discs, SMOD) against the average of these values. The mean differences are indicated as dashed horizontal lines, and limits of agreement (95% confidence interval) are indicated as continuous horizontal lines. A: Bland-Altman analysis evaluating the concordance between biplane ALM and SMOD for assessing LADvol/BW. B: Bland-Altman analysis evaluating the concordance between between biplane ALM and SMOD for assessing LASvol/BW. C: Bland-Altman analysis evaluating the concordance between monoplane ALM and SMOD for assessing LADvol/BW. D: Bland-Altman analysis evaluating the concordance between monoplane ALM and SMOD for assessing LASvol/BW.

**Fig 5:**
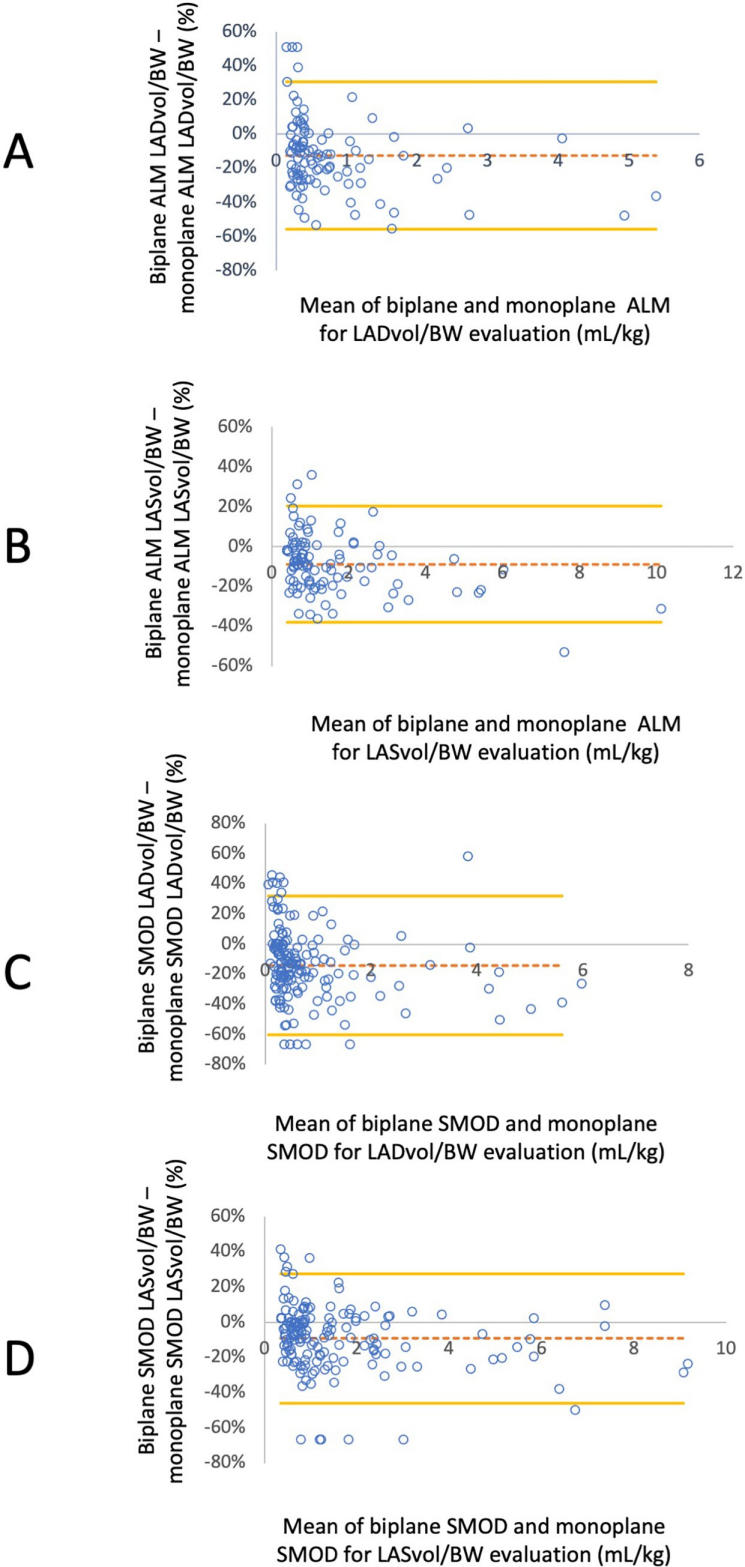
Bland-Altman analysis illustrating the difference between end-diastolic and end-systolic left atrial volumes normalized to body weight (LADvol/BW and LASvol/BW, respectively) assessed by different echocardiographic methods (i.e., monoplane and biplane area length methods (ALM) and Simpson’s methods of discs, SMOD) against the average of these values. The mean differences are indicated as dashed horizontal lines, and limits of agreement (95% confidence interval) are indicated as continuous horizontal lines. A: Bland-Altman analysis evaluating the concordance between biplane and monoplane ALM for assessing LADvol/BW. B: Bland-Altman analysis evaluating the concordance between biplane and monoplane ALM for assessing LASvol/BW.

**Table 3:** Results from Bland and Altman analyses evaluating differences between left atrial volumes assessed by different echocardiographic methods.

Left atrial volume assessment method	Bias (95% confidence interval)	95% limits of agreement
**LADvol/BW**	8.2%	- 37%; + 53,5%
*Biplane ALM versus biplane SMOD*	(3.7; 12.8%)	
**LASvol/BW**	7.3%	- 6.4%; + 21%
*Biplane ALM versus biplane SMOD*	(5.9; 8.7%)	
**LADvol/BW**	9.5%	- 8.5; + 27.6%
*Monoplane ALM versus monoplane SMOD*	(7.8; 11.3%)	
**LASvol/BW**	8.5%	- 4.4%; + 21.4%
*Monoplane ALM versus monoplane SMOD*	(7.3; 9.8%)	
**LADvol/BW**	- 12.8%	- 55.9%; + 30.4%
*Biplane ALM versus monoplane ALM*	(-17.1; -8.4%)	
**LASvol/BW**	- 8.7%	- 38.1%; + 20.6%
*Biplane ALM versus monoplane ALM*	(-11.7; -5.8%)	
**LADvol/BW**	- 14.1%	- 60.3%; + 32.1%
*Biplane SMOD versus monoplane SMOD*	(-17.8; -10.4%)	
**LASvol/BW**	- 9.2%	- 46.0%; + 27.7%
*Biplane SMOD versus monoplane SMOD*	(-12.1; -6.2%)	

ALM: area length method. LASvol/BW: end-systolic left atrial volume normalized to body weight. LADvol/BW: end-diastolic left atrial volume normalized to body weight. SMOD: Simpson’s method of discs.

#### Repeatability and inter-observer reproducibility of LA:Ao ratios and LA volumes

**Tables 4** and **5** present Lin’s CCC as well as bias and limits of agreements from Bland and Altman’s analyses regarding repeatability and inter-observer reproducibility of end-systolic and end-diastolic LA:Ao ratios and of LASVol/BW and LADVol/BW. All Lin’s CCCs were considered as very good to excellent, except for end-diastolic LA:Ao ratio (fairly good). Limits of agreement were considered as good considering maximum expected values a priori defined by the clinicians.

**Table 4:** Repeatability expressed as Lin’s concordance correlation coefficient (CCC) as well as 95% limits of agreement obtained by Bland and Altman analyses regarding end-diastolic and end-systolic left atrial volumes indexed to body weight (LADvol/BW and LASvol/BW, respectively) assessed by different echocardiographic methods (i.e., monoplane and biplane Simpson’s methods of discs (SMOD) and area length methods (ALM)) and left atrium-to-aorta ratios (Observer 1).

	Lin’s CCC (95% confidence interval)	95% limits of agreement
**Left atrial volumes**		
*LASvol/BW (SMOD*, *monoplane)*	0.99 (0.99–1.0)	-0.08; 0.07 mL/kg
*LASVol/BW (ALM*, *monoplane)*	1.0 (0.99–1.0)	-0.1; 0.07 mL/kg
*LADVol/BW (SMOD*, *monoplane)*	0.99 (0.99–1.0)	-0.04; 0.05 mL/kg
*LADVol/BW (ALM*, *monoplane)*	0.99 (0.99–1.0)	-0.05; 0.06 mL/kg
*LASvol/BW (SMOD*, *biplane)*	1.0 (0.99–1.0)	-0.05; 0.05 mL/kg
*LASVol/BW (ALM*, *biplane)*	1.0 (0.99–1.0)	-0.07; 0.05 mL/kg
*LADVol/BW (SMOD*, *biplane)*	1.0 (0.99–1.0)	-0.03; 0.04 mL/kg
*LADVol/BW (ALM*, *biplane)*	1.0 (0.99–1.0)	-0.03; 0.04 mL/kg
***Left atrium*:*aorta ratios***		
*End-diastolic left atrium*:*aorta ratio*	0.97 (0.92–0.99)	-0.07; 0.07
*End-systolic left atrium*:*aorta ratio*	0.98 (0.96–0.99)	-0.08; 0.07

**Table 5:** Inter-observer reproducibility expressed as Lin’s concordance correlation coefficient (CCC) as well as bias and 95% limits of agreement obtained by Bland and Altman analyses regarding end-diastolic and end-systolic left atrial volumes indexed to body weight (LADvol/BW and LASvol/BW, respectively) assessed by different echocardiographic methods (i.e., monoplane and biplane Simpson’s methods of discs (SMOD) and area length methods (ALM)) and left atrium-to-aorta ratios (Observer 2 *versus* Observer 1).

	Lin’s CCC (95% confidence interval)	Bias (95% confidence interval)	95% limits of agreement
**Left atrial volumes**			
*LASvol/BW (SMOD*, *monoplane)*	0.97 (0.94–0.99)	0.03 (-0.07; 0.01) mL/kg	-0.22; 0.15 mL/kg
*LASVol/BW (ALM*, *monoplane)*	0.99 (0.96–0.99)	0.02 (-0.01; 0.05) mL/kg	-0.13; 0.17 mL/kg
*LADVol/BW (SMOD*, *monoplane)*	0.97 (0.94–0.99)	0 (-0.02; 0.02) mL/kg	-0.11; 0.11 mL/kg
*LADVol/BW (ALM*, *monoplane)*	0.98 (0.95–0.99)	0.02 (0; 0.03) mL/kg	-0.08; 0.11 mL/kg
*LASvol/BW (SMOD*, *biplane)*	0.98 (0.95–0.99)	-0.04 (-0.06; -0.01) mL/kg	-0.17; 0.09 mL/kg
*LASVol/BW (ALM*, *biplane)*	0.99 (0.98–1.0)	0 (-0.02; 0.02) mL/kg	-0.1; 0.1 mL/kg
*LADVol/BW (SMOD*, *biplane)*	0.99 (0.97–0.99)	0 (-0.01; 0.01) mL/kg	-0.06; 0.06 mL/kg
*LADVol/BW (ALM*, *biplane)*	0.98 (0.96–0.99)	0.01 (0; 0.03) mL/kg	-0.06; 0.09 mL/kg
***Left atrium*:*aorta ratios***			
*End-diastolic left atrium*:*aorta ratio*	0.86 (0.71–0.94)	0 (-0.02; 0.03)	-0.12; 0.12
*End-systolic left atrium*:*aorta ratio*	0.93 (0.85–0.97)	0.01 (-0.01; 0.04)	-0.12; 0.15

## Discussion

Early identification of CKCSs with subclinical DMVD and heart remodeling is of great practical interest, in regard to clinical staging, breeding recommendation, risk assessment, therapeutical decision, and prognosis [21, 35]. Chronic and hemodynamically significant MR related to DMVD results in left heart volume overload, firstly characterized by LA enlargement as commonly evaluated by the LA:Ao ratio [9], which is one of the most significant predictors of poor outcome and cardiac related death in DMVD dogs [11, 36]. However, echocardiographic identification of mild left heart enlargement including LA dilation can be challenging [8]. Volume-based echocardiographic methods for measuring LA size seem relevant, because LA enlargement may occur asymmetrically in various directions. Several methods for assessing LA volume using 2D mode have been evaluated in dogs [15–21]. To the best of the authors’ knowledge, the present prospective study provides original data regarding monoplane and biplane SMOD and ALM in LA volume evaluation in a large population of CKCSs, and also compares the ability of LA volumes and LA:Ao ratios to identify LA enlargement in ACVIM stage B DMVD dogs.

The present study shows that LA volume measurements are adequate for clinical routine use in the CKCS breed, as all Lin’s CCCs regarding repeatability and inter-observer reproducibility were considered as very good to excellent for LASVol/BW and LADVol/BW with whatever the method used, i.e., monoplane and biplane SMOD and ALM (0.97 to 1.0). Additionally, regarding LA volumes indexed to BW, limits of agreement obtained by Bland and Altman analyses were also considered as good using the *a priori* defined cut-off value (i.e., <0.30 mL/kg), the maximum limits of agreement for repeatability and inter-observer reproducibility respectively being -0.08 mL/g and -0.22 mL/kg. These results are in accordance with past studies that also showed good to high repeatability and reproducibility of biplane and monoplane methods for assessing LA volume in dogs [15–17], although some authors demonstrated that volume measurements exhibit more within-day, between-day, and interoperator variability than did linear estimate measurements [37].

In the present study, both monoplane methods produced higher LA volume measurements (i.e., LASvol/BW and LADvol/BW) by comparison with both biplane methods, with ALM also leading to higher LA volumes by comparison with SMOD. Therefore, echocardiographic methods for assessing LA volumes in the CKCS breed are not interchangeable, and the same method should be used for longitudinal follow-up of a given dog from this specific breed. The discrepancy between monoplane and biplane measurements has already been reported by other authors [17]. It may be explained by the fact that the LA cavity is not hemispheric, having larger dimensions on the left apical 4-chamber view than the apical 2-chamber view [17]. Regarding SMOD and ALM, the present results are also in accordance with previous studies, which included healthy dogs and dogs with DMVD from different breeds and demonstrated that SMOD provides lower values of LA volumes than ALM, with SMOD underestimating and ALM overestimating LA volumes as compared to real-time 3-dimensional echocardiographic methods [17, 20]. Additionally, differences between SMOD and ALM have been shown to increase with expansion of the LA size [20] and may be explained by the different geometric assumptions made for ALM and SMOD volume calculations, in which ALM considers LA as an elliptic chamber and SMOD as a cylinder [17]. Comparison between the two 2D echocardiographic methods (SMOD and ALM) and real-time 3-dimensional echocardiography demonstrated that biplane SMOD, and not ALM, would be the 2D echocardiographic method of choice for assessing LA volumes in the dog, since a mean underestimation of only 7% was found for SMOD *versus* a mean overestimation of 24% for ALM [20].

In the present study, similarly to LA:Ao ratios, whatever method used (i.e., monoplane and biplane ALM and SMOD), both LASvol/BW and LADvol/BW were significantly correlated with echocardiographic parameters known to reflect DMVD severity, i.e., RF assessed by the PISA method, TRPG, and mitral E velocity [11, 12, 36]. Similarly, a significant difference in EF_LA_ was observed between ACVIM stages with EF_LA_ decreasing with increasing DMVD severity and with a negative correlation between RF and EF_LA_. Such results are consistent with those from other reports showing a strong correlation between EF_LA_ and canine DMVD severity related to decreased LA contractility and increased LA afterload [38, 39]. Additionally, regarding ACVIM stage B1, CKCSs with mild MR characterized by RF values ≤30% showed significantly lower LASvol/BW (assessed monoplane and biplane ALM and SMOD) than CKCSs with RF values >30%. Interestingly, in one study, among dogs with subclinical DMVD and normal LA:Ao ratio, significantly higher plasma N-terminal pro-B-type natriuretic peptide concentrations were found in those with RF >30% than in those with RF ≤30% [40]. One may hypothesize that these higher cardiac biomarker concentrations were actually related to a mild LA enlargement, which was unapparent using linear LA measurement.

Regarding LA and LV dilation criteria, there is now scientific evidence that the proposed multibreed echocardiographic ACVIM cut-offs [8] do not perfectly fit for several specific breeds, and the use of breed-specific reference intervals would be more appropriate [41, 42]. As recently demonstrated by Rishniw and Brown [41], approximately 10% of healthy CKCSs would be misclassified as having LV enlargement using the ACVIM-recommended scaling exponent (0.294) and the cut-off of 1.7 for normalized end-diastolic LV internal diameter. The study published by Misbach et al dedicated to the establishment of echocardiographic reference intervals in a large population of healthy CKCSs also demonstrated the discrepancy between their LV breed-specific reference intervals (assessed according to the Clinical Laboratory and Standard Institute recommendations) and the predictive reference intervals obtained in the same population using Cornell’s formula [23].

Regarding LA size, CKCS dogs seem to have smaller end-diastolic and end-systolic LA dimensions than most other breeds [14, 15, 23]. In the present study, LA/Ao ratios were measured at end-diastole and end-systole as respectively described in CKCSs by Misbach et al [23] and Hansson et al [14]. End-diastolic LA:Ao values of our ACVIM stage A CKCS dogs (Table 1) are consistent with reference intervals (lower and upper limits with 90% confidence intervals) from Misbach et al, i.e., 0.54 (0.47–0.56) - 0.93 (0.90–0.94) [23]. Similarly end-systolic LA:Ao values of our ACVIM stage A CKCS dogs (Table 1) are consistent with those from Hansson et al [14] who reported relatively low LA:Ao ratios at end-systole on 56 healthy CKCSs, with mean ± SD (range) values of 1.03 ± 0.09 (0.84–1.27). Based on these data, the end-systolic LA:Ao ratio cut-off value of 1.6 proposed by the 2019 consensus ACVIM statement to detect end-systolic LA enlargement in all-breed dogs appears inappropriate (i.e., too high) for CKCSs, with a risk of missing some mild LA dilations.

Previous studies suggested that LA volumes might possibly be better than LA:Ao ratios for identifying LA enlargement in dogs with DMVD [16, 17]. The present prospective study demonstrates that no dogs with increased LA:Ao ratios had normal LA volumes. Conversely, a sub-population of ACVIM B1 dogs with normal LA/Ao ratios actually showed LA dilation detected by LA volume calculation, which was not apparent when applying end-diastolic and end-systolic LA:Ao ratios. This confirms that LA volume quantification is more effective to detect LA dilation than linear LA measurements. From a practical point of view, this also suggests that considering linear LA measurements only, may lead to an erroneous left heart evaluation, which will not be the case when using volumetric methods only. The high negative prognostic value of LA enlargement has been demonstrated in DMVD dogs [11, 37, 38], with LA volume being the strongest predictor of cardiac-related death, highly superior to all other parameters of LA size and function including LA/Ao ratios [43, 44]. Therefore, as in human cardiology [45], LA volumetric quantification should the method of first choice for assessing LA size particularly to stratify ACVIM stage B dogs. Additionally, the early identification of LA enlargement in stage B dogs is of practical interest, as these dogs at risk for decompensation may benefit from a closer longitudinal follow-up than others. Additionally, among the 31 CKCSs with normal end-systolic LA:Ao ratio but increased LASvol/BW volume (**Fig 3**), 19/31 (61%) showed increased end-diastolic LV diameter [23]. In other words, these 19 dogs were characterized by mitral valve regurgitation severe enough to have induced both LA and LV dilation (with a median RF value of 38%). Thus, according to the 2019 ACVIM classification [8], these dogs may actually belong to ACVIM stage B2.

This study presents several limitations. Firstly, treatment of DMVD dogs at inclusion may have exerted an influence on LA volumes and LA:Ao ratios to an unknown extent. Secondly, the subpopulation of healthy CKCSs (ACVIM stage A dogs) was lower than that of CKCSs affected by DMVD, and further studies in large populations of healthy CKCSs are needed to establish reference intervals for LA volumes in this canine breed.

Moreover, LA volumes assessed by 2D echocardiographic methods were not compared to gold standard techniques (i.e., cardiac magnetic resonance imaging and ECG-gated multidetector computed tomography angiography) or even to 3-dimensional echocardiography, which has been demonstrated to be more precisely correlated with computed tomography than 2D echocardiographic methods [46, 47]. They were neither compared to a relevant DMVD outcome. However, when compared with gold standard techniques, 2D echocardiographic methods appear reliable in evaluating LA volume, with an acceptable estimation of measured volumes hence requiring less specific and expensive material and being less time-consuming, without implementing general anesthesia [46, 47]. Besides, LA volumes and LA:Ao ratios were assessed using one single 2D measurement for each value. For biplane ALM, LA volumes were calculated by averaging the LA volumes assessed from the left apical 4- and 2-chamber views. Various biplane ALM formula are used in human and veterinary cardiology [15, 16, 45]. In human cardiology, the following formula is commonly used [45]: [0.85 x A1 x A2 / LA length], with A1 and A2 being the LA area from apical 4- and 2-chamber views, and LA length being defined as the shortest of the two long axes measured. However, to provide reliable calculations the two LA lengths should not differ more than 5 mm [45], which represents also a limitation of this method. Additionally, the repeatability results presented here are only valid for the two involved observers and the assessment of repeatability was performed on recorded loops (and not on healthy or diseased animals), which might have underestimated the actual measurement variability. Investigating the impact of arrhythmias was beyond the scope of the present study. This represents another limitation, as arrhythmias like atrial fibrillation, though uncommon in CKCSs with DMVD, have been shown to be associated with more advanced stages of the disease and increased LA dimensions [48]. Lastly, in the present study, the ACVIM guidelines criteria for defining stage B2 were purposefully partially used, as CKCS breed-specific echocardiographic cut-offs were chosen according to published data [14, 23, 41, 42]. The present results are therefore only valid with these breed-specific cut-offs. Whether similar conclusions could be drawn with the ACVIM multi-breed generic cut-offs for LA and LV enlargement should be addressed by further studies.

In conclusion, these results demonstrate the practical interest of LA volume assessment in CKCSs with DMVD, especially among asymptomatic dogs, as a proportion of B1 dogs demonstrated volumetric LA enlargement despite having a normal LA:Ao ratio according to specific CKCS breed reference intervals. Further studies are now needed to determine the prognostic value of LA volume in CKCSs with DMVD.

## Supporting information

S1 File (DOCX)

S1 Data (XLSX)
