# Supplementary material for: Volumetric quantification identifies some left atrial dilations undetected by left atrium:aorta ratio measurements: A prospective echocardiographic study in 155 Cavalier King Charles Spaniels with and without degenerative mitral valve disease
Source: PLoS One. 2024 Mar 28;19(3):e0300827. doi: 10.1371/journal.pone.0300827 (PMC10977888; doi:10.1371/journal.pone.0300827)
Supplement: S1 File — (DOCX) [file pone.0300827.s001.docx]

|  | MINISTERE DE L'AGRICULTURE  ECOLE NATIONALE VETERINAIRE d’ALFORT  **Unité de Cardiologie d’Alfort**  **UMR de cardiologie INSERM-ENVA U955**  Département d'Elevage, de Pathologie des Equidés et Carnivores  7, avenue du Général de Gaulle  94700 MAISONS-ALFORT - France |  |
| --- | --- | --- |

##### February 15, 2024

Dear Editor,

This letter is to confirm that concomitantly with the submitted second revised version of our manuscript PONE-D-23-22610 (entitled “*Volumetric quantification identifies some left atrial dilations undetected by left atrium:aorta ratio measurements: a prospective echocardiographic study in 155 Cavalier King Charles Spaniels with and without degenerative mitral valve disease*”), we did upload all our raw data (i.e., our entire data set from the 155 Cavalier King Charles included in the study) as a supplementary xls file.

These data include all the values used in the present article for 1) descriptive and statistical analyses, 2) Tables, and also 3) Figures. It includes even more, e.g., dogs’ and owners’ name together with files numbers and exam dates.

Sincerely yours,

Professor Valérie Chetboul

DVM, PhD, ECVIM-CA (Cardiology) diplomate

Tel: 00 33 6 12 11 43 87 - E-mail: [vchetboul@vet-alfort.fr](mailto:vchetboul@vet-alfort.fr)
